# Efficiency in sequential testing: Comparing the sequential probability ratio test and the sequential Bayes factor test

**DOI:** 10.3758/s13428-021-01754-8

**Published:** 2022-03-01

**Authors:** Angelika M. Stefan, Felix D. Schönbrodt, Nathan J. Evans, Eric-Jan Wagenmakers

**Affiliations:** 1 Department of Psychology, University of Amsterdam, Amsterdam, The Netherlands; 2 Department of Psychology, Ludwig-Maximilians-Universität München, München, Germany; 3 School of Psychology, University of Queensland, St Lucia, Australia

**Keywords:** Bayesian inference, Likelihood tests, Sample size determination, Bayes factor design analysis, Experimental design, Statistical error control, Design optimization, Hypothesis testing

## Abstract

In a sequential hypothesis test, the analyst checks at multiple steps during data collection whether sufficient evidence has accrued to make a decision about the tested hypotheses. As soon as sufficient information has been obtained, data collection is terminated. Here, we compare two sequential hypothesis testing procedures that have recently been proposed for use in psychological research: Sequential Probability Ratio Test (SPRT; *Psychological Methods, 25*(2), 206–226, 2020) and the Sequential Bayes Factor Test (SBFT; *Psychological Methods, 22*(2), 322–339, 2017). We show that although the two methods have different philosophical roots, they share many similarities and can even be mathematically regarded as two instances of an overarching hypothesis testing framework. We demonstrate that the two methods use the same mechanisms for evidence monitoring and error control, and that differences in efficiency between the methods depend on the exact specification of the statistical models involved, as well as on the population truth. Our simulations indicate that when deciding on a sequential design within a unified sequential testing framework, researchers need to balance the needs of test efficiency, robustness against model misspecification, and appropriate uncertainty quantification. We provide guidance for navigating these design decisions based on individual preferences and simulation-based design analyses.

Across scientific disciplines, researchers use statistical hypothesis tests to evaluate the outcomes of experiments that assess the validity of claims about the world. Conducting scientific experiments can require substantial resources of time, money, and effort, and can put human and animal subjects under considerable strain. It is therefore in the best interest of all scientific stakeholders to use efficient hypothesis testing procedures (Hunter & Hoff, 1967). Since the costs of a study are often proportional to sample size (Dupont & Plummer, 1990), optimizing research efficiency means finding a research design that minimizes the number of observations needed (Myung & Pitt, 2009).

Sequential designs constitute a powerful tool to achieve experimental efficiency (e.g., Wald & Wolfowitz 1948; Schönbrodt et al., 2017). On average, sequential hypothesis tests yield substantially smaller sample sizes than conventional hypothesis tests that assume a fixed sample size based on a statistical power analysis. Studies investigating the efficiency of sequential designs have consistently found reductions in sample sizes of 50% or more compared to these fixed sample size designs (e.g., Wald 1945; Schönbrodt et al., 2017; Schnuerch & Erdfelder 2020). This makes sequential hypothesis testing an attractive choice when resources are scarce.

In a sequential hypothesis test, researchers check at every step of the data collection process whether sufficient evidence has been obtained to make a decision about the tested hypotheses.^1^ Data collection is terminated as soon as sufficient information about the tested hypotheses has accrued (Wald, 1945). Conceptually, this sequential sampling procedure resembles the practice of “optional stopping” that has been repeatedly criticized as a questionable research practice (Armitage et al., 1969; John et al., 2012). However, sequential hypothesis tests provide a framework of carefully designed decision rules that allow for valid statistical inference despite optional stopping (Wald, 1945).

Recently, two existing sequential hypothesis testing methods have been brought to the attention of a wide audience of psychology researchers: the Sequential Probability Ratio Test (SPRT; Schnuerch & Erdfelder 2020) and the Sequential Bayes Factor Test (SBFT; Schönbrodt et al., 2017). Both methods are applicable to a wide range of hypothesis tests, and can therefore be used in many research scenarios (Wald, 1945; Rouder, 2014).

In some disciplines of psychology, the SPRT has already enjoyed great popularity for many years. In psychological assessment, Ferguson designed a sequential mastery test using the SPRT methodology as early as 1969. Seminal work by Reckase (1983) combined the SPRT with item response theory, and has been the basis for more recent methodological developments in the field of computerized classification testing (e.g., Lin and Spray 2000; Eggen & Straetmans 2000; Eggen 2011; Finkelman 2008). The SPRT has also been used to study speeded decision making in humans and other animals (Luce, 1986; Townsend & Ashby, 1983; Bogacz et al., 2006; Ratcliff, 1978; Purcell et al., 2010; Milosavljevic et al., 2010), and is the basis for modern models of human decision making such as the Drift Diffusion Model (Bogacz et al., 2006; Griffith et al., 2021). With the increasing uptake of Bayesian statistics in psychology in the past years, SBFTs have become more popular as well. For example, Bayesian sequential testing has been applied in developmental psychology in experiments on early word learning (Mani et al., 2020), or in cognitive psychology in experiments on learning and decision making (Perquin et al., 2020; Stojić et al., 2020). Recently, there has been a strong focus on developing, comparing, and promoting sequential testing procedures for the independent-samples *t*-test, one of the most commonly used statistical tests in psychological research (Wetzels et al., 2011). Our paper will use the *t*-test as an example as well, but our results apply to the broader class of SPRTs and SBFTs.

In a recent article, Schnuerch and Erdfelder (2020) contrasted the SPRT *t*-test and the sequential Bayesian *t*-test based on simulations with diffuse priors and a specific set of stopping rules. For this scenario, they concluded that the two main advantages of the SPRT are that (1) it is more efficient than the sequential Bayesian test, and (2) it allows for explicit control of error rates. A similar claim was made by Pramanik et al., (2020) based on their comparison of the SBFT and a modified SPRT procedure that allows researchers to set a maximum sample size.

Here, we demonstrate that the simulation conditions used by Schnuerch and Erdfelder (2020) constitute an extreme case within an otherwise more nuanced relationship between the two hypothesis testing procedures. Specifically, we extend their simulations to show that the SPRT and SBFT are procedurally and mathematically similar, and that their relative efficiency depends on the exact model specification and the true population parameters. We argue that recent attempts to compare the efficiency of the two methods have presented favorable scenarios for the SPRT, as the true effect sizes perfectly matched the effect size assumptions postulated in its statistical models. We also demonstrate that simulation and optimization techniques make explicit error control possible for both hypothesis testing procedures. Given the close relationship between the SPRT and SBFT, we argue that the choice of the sequential testing method can be regarded as a multi-dimensional choice within a unified framework where several desiderata can be weighted with regard to the specific research context at hand. This presents a new perspective because although previous research has acknowledged the similarities between the two sequential testing methods (e.g., Schnuerch & Erdfelder 2020), authors have typically treated them as distinct methodologies. We believe that there are many theoretical and practical advantages to viewing them as part of an overarching framework, and will present these in the course of our paper.

Our manuscript is structured as follows. First, we will show that the SPRT and SBFT can be regarded as two instances of a common sequential hypothesis testing framework. Then, we explore what this means for the efficiency of the two hypothesis testing methods using a set of simulation studies. We show that differences in efficiency between the two hypothesis testing procedures are gradual and depend on the exact model specification. The third part of our manuscript addresses the question of choosing an adequate sequential hypothesis testing method in an applied research setting. Based on our simulations, we discuss several desiderata that researchers need to weigh in planning sequential designs, and present several pragmatic research strategies that result from these design decisions.

## How similar are the SBFT and SPRT?

Previous research has presented the SBFT and SPRT as two distinct methodologies for sequential hypothesis testing (e.g., Schnuerch & Erdfelder 2020; Pramanik et al., 2020). In the following sections, we will demonstrate that the methods are, in fact, part of the same overarching hypothesis testing framework. Our argument rests two pillars: First, we show that the monitored outcome that quantifies the collected evidence at every step of the sequential process, is closely related in the SPRT and SBFT. Second, we demonstrate that stopping rules can be defined based on the same principles for both sequential testing procedures. Since the error rates of a sequential hypothesis test are governed by the definition of the stopping rule, we also show that error rates can be explicitly controlled in both procedures.

### Evidence monitoring: Likelihood ratio vs. Bayes factor

All sequential hypothesis tests are based on monitoring an analysis outcome as sample size increases. If the outcome fulfills certain pre-specified stopping criteria, data collection is terminated. If it does not fulfill the criteria, data collection is continued by collecting an additional observation (Wald, 1945; Schönbrodt et al., 2017). Both the SPRT and the SBFT monitor a quantity that measures the relative evidence for two competing models, with the models representing the null and the alternative hypothesis, respectively. As soon as sufficient evidence for either model has accrued, the data collection is stopped and a decision is made in favor of the model that received stronger support from the data (Wald, 1945).

#### SPRT: Monitoring the likelihood ratio

In the SPRT, the monitored quantity is a likelihood ratio that is defined as the likelihood of the data *D* under the alternative model ${\mathscr{M}}_{1}$ divided by the likelihood of the data under the null model ${\mathscr{M}}_{0}$,

1 $$ \text{LR}_{10} = \frac{f(D \mid \mathcal{M}_{1})}{f(D \mid \mathcal{M}_{0})} = \frac{f(D \mid \boldsymbol{\theta}_{1})}{f(D \mid \boldsymbol{\theta}_{0})} . $$

Each model likelihood function contains a set of fixed parameter values, ***𝜃***_*i*_, with *i* ∈{0,1}.

For example, in the one-sided SPRT *t*-test, the likelihood functions for the null and alternative model are both *t*-distributions with *ν* = *n*_1_ + *n*_2_ − 2 degrees of freedom, where *n*_1_ and *n*_2_ are the sample sizes of the two groups that should be compared.^2^ The data are summarized by a *t*-statistic (Rushton, 1950; Schnuerch and Erdfelder, 2020). The null model posits that Cohen’s *δ*_0_, the standardized mean difference between the groups, equals zero. The alternative model assumes a fixed effect size *δ*_1_ that differs from zero. The non-centrality parameter Δ_*i*_ of the *t*-distributions depends on the effect size that is assumed in the models,

2 $$ \begin{array}{@{}rcl@{}} &&\text{LR}_{\text{SPRT \textit{t}-test, one-sided}} = \frac{f(t\mid \nu, {\Delta}_{1}) }{f(t\mid \nu, {\Delta}_{0})},\\ &&\text{with } {\Delta}_{i} = \delta_{i}  \sqrt{\frac{n_{1} \times n_{2}}{n_{1} + n_{2}}} \text{ for } i = \{0, 1\} . \end{array} $$

In the two-sided SPRT *t*-test (Hajnal, 1961), the likelihood functions are based on squared *t*-values to indicate that no knowledge about the sign of the effect exists. Since it can be shown that *t*^2^(*ν*,Δ) = *F*(1,*ν*,Δ^2^), the likelihood ratio can be expressed as the ratio of a noncentral to a central *F*-distribution (Brereton, 2015),

3 $$ \text{LR}_{\text{SPRT \textit{t}-test, two-sided}} = \frac{f(t^{2} \mid \nu, {\Delta}_{1})}{f(t^{2} \mid \nu, {\Delta}_{0})} = \frac{f(F \mid 1, \nu, {{\Delta}_{1}^{2}})}{f(F \mid 1, \nu, {{\Delta}_{0}^{2}})} . $$

Note that the quantity monitored in the SPRT differs from the test statistic in the generalized likelihood ratio test (GLR; Neyman & Pearson 1928). Specifically, the likelihood functions are evaluated at fixed parameter values, and not at their data-dependent maximum. This is also true for the sequential extensions of the GLR test, as developed by Li et al., (2014), or by Thompson (2009) for computerized adaptive testing.

#### SBFT: Monitoring the Bayes factor

In the SBFT, the monitored analysis outcome is the Bayes factor. Conceptually, the Bayes factor can be understood as an extension of the likelihood ratio that accounts for uncertainty about the model parameters (Jeffreys, 1961). This epistemic uncertainty is expressed through the prior distribution, that is, a probability density function that is placed on parameter values in the statistical model. Mathematically, the Bayes factor is defined as the ratio of two marginal likelihoods (Kass and Raftery, 1995), in which the likelihood function, *f*(*D*∣***𝜃***), is weighted by the prior distribution, *p*(***𝜃***), and averaged across the parameter space. The Bayes factor can therefore be defined as

4 $$ \text{BF}_{10} = \frac{p(D \mid \mathcal{M}_{1})}{p(D \mid \mathcal{M}_{0})} = \frac{\int f(D \mid \boldsymbol{\theta}_{1})  p(\boldsymbol{\theta}_{1}) \text{d}\boldsymbol{\theta}_{1}}{\int f(D \mid \boldsymbol{\theta}_{0})  p(\boldsymbol{\theta}_{0})  \text{d}\boldsymbol{\theta}_{0}} . $$

The specification of the prior distribution has been a longstanding topic of discussion among Bayesian statisticians (e.g., Jeffreys 1961; Goldstein 2006; Berger 2006; Lindley 2004). Researchers often use so-called “uninformative” or “default” prior distributions for the alternative model (Berger, 2006). These prior distributions are specified to fulfill several conceptual desiderata and assign non-negligible probabilities to a wide range of values (Consonni et al., 2018). A common default prior distribution for the effect size parameter *δ* in the alternative model in a Bayesian *t*-test is a zero-centered Cauchy distribution with a scale parameter of $\sqrt {2}/2$ (Rouder et al., 2009; Morey & Rouder, 2018). In this distribution, 50% of the probability mass lies between values of − 0.707 and + 0.707. In the remainder of this article, we refer to this prior specification as the default prior setup.

An alternative to default prior distributions are informed prior distributions that incorporate substantive application-specific prior knowledge about the parameter (Goldstein, 2006). As a general rule, informed prior distributions are more peaked around certain parameter values when more prior knowledge exists (Dienes, 2019). In the most extreme case, the prior distribution can be reduced to a point prior that assigns all mass to a single value (Etz et al., 2018). Informed prior distributions can be defined based on theoretical considerations, previous literature, or expert knowledge (Vanpaemel, 2010; Verhagen & Wagenmakers, 2014; Stefan et al., 2020). In Bayesian *t*-tests, informed prior distributions on the effect size *δ* in the alternative model most often take the form of a non-central normal or *t*-distribution (Gronau et al., 2020). One particular piece of information that can be integrated in the prior distribution is the information about the sidedness of the effect. For example, the facial feedback hypothesis posits a one-sided effect, namely that participants who hold a pen between their teeth rate a cartoon as *more* funny than participants who hold the pen between their lips (Strack et al., 1988). For positive directional one-sided tests like this, the prior distribution on the parameter of interest is truncated to include only positive values (Wagenmakers et al., 2010).

#### Bayes factors converge to likelihood ratios

**Fig. 1:**
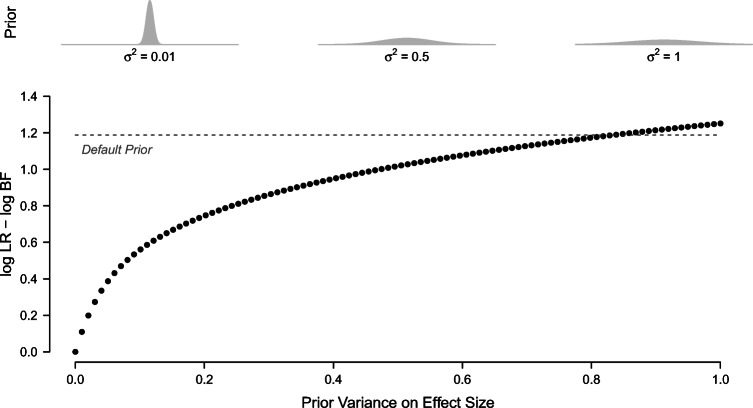
Difference between the log likelihood ratio and the log Bayes factor for a *t*-value of 2.5, i.e., a sample effect size of *δ*= 0.5 in a sample of size 50 per group. The mode of the prior distribution and the effect size assumed in the alternative model in the likelihood ratio are equal to *δ*_1_ = 0.5. The Bayes factor approaches the likelihood ratio as the width of the prior distribution decreases

It is important to note that if point priors are placed on all model parameters, the Bayes factor reduces to a likelihood ratio (Jeffreys 1961, p. 396). In Bayesian null hypothesis testing, it is customary to specify a point prior on zero for the parameter of interest in the null hypothesis and a default or informed prior distribution for the parameter of interest in the alternative model (Wagenmakers et al., 2010). Therefore, as the width of the prior distribution around a parameter value in the alternative model decreases, the Bayes factor approaches the likelihood ratio that specifies the same parameter value in the alternative model. Figure 1 demonstrates this in an example for the *t*-test. In the example, the Bayes factor uses a normal distribution centered on Cohen’s *δ* of 0.5 as a prior distribution on effect size in the alternative model. The alternative model in the likelihood ratio also assumes an effect size of *δ*_1_ = 0.5. As the variance of the normal prior decreases, the difference between the log Bayes factor and the log likelihood ratio for the same data decreases as well.

#### Implications for sequential testing

For sequential tests, the relationship between likelihood ratios and Bayes factors implies that the monitored outcome in the SPRT and in the SBFT can be numerically very close. The degree of similarity depends on the extent to which the SPRT parameter value is representative of the prior distribution in the Bayesian test. Although the effect of informed prior distributions on Bayesian sequential testing has been demonstrated earlier (Stefan et al., 2019), recent efforts to compare the SPRT and SBFT have focused solely on sequential Bayesian tests with default prior distributions (Schnuerch & Erdfelder, 2020; Pramanik et al., 2020). However, as becomes clear from Fig. 1, this comparison provides an extreme example of the differences that can occur between the SPRT and sequential Bayesian tests. The reason for this is that default prior distributions are not only relatively wide, but their mode also does not coincide with the parameter value in the alternative model in the SPRT. Notably, the specification of uncertainty about parameters allows for a seamless integration of model comparison and parameter estimation in the Bayesian framework, since prior distributions can be meaningfully updated to posterior distributions. Thus, although the Bayes factor is numerically nearly identical to the likelihood ratio if the prior is extremely narrow, there remains a qualitative difference in the possibility of the model to incorporate new information. We will discuss this in more detail in the section about uncertainty specification later in this paper.

### Stopping rules and error rate control

The sequential testing procedure in the SPRT and SBFT requires the definition of an upper and lower evidence threshold (Wald, 1945; Schönbrodt et al., 2017). If the outcome measure is smaller than the lower threshold, a decision for the null hypothesis is made; if the outcome measure is larger than the upper threshold, a decision for the alternative hypothesis is made; if the outcome measure lies between the two thresholds, an additional observation is collected.

The threshold definition is directly related to the error rates and the average sample size of the sequential design. Generally, wider thresholds lead to higher average sample sizes because more evidence is required to make a decision. However, wider thresholds also lead to lower rates of false-positive and false-negative decisions (Schnuerch & Erdfelder, 2020; Stefan et al., 2019). This makes the definition of thresholds for sequential designs an interesting optimization problem. In the following, we show that the definition of optimal thresholds follows the same principles in the SPRT and SBFT.

#### SPRT: Controlling error rates with Wald’s thresholds

For the SPRT, Wald (1945) recommended constant thresholds defined through the following formulae,

5 $$ \begin{array}{@{}rcl@{}} A &=& \frac{(1-\beta)}{\alpha}  ,\\ B &=& \frac{\beta}{(1-\alpha)}  , \end{array} $$

where A and B are the values of the two thresholds on the likelihood ratio, and *α* and *β* are the maximum rates of false-positive evidence and false-negative evidence, respectively, that a researcher is willing to tolerate. For example, a researcher aiming for nominal error rates of *α* = 0.05 and *β* = 0.1 would calculate Wald’s thresholds as *A* = (1 − 0.1)/0.05 = 18, and *B* = 0.1/(1 − 0.05) = 0.105.

It can be shown that if either of the postulated models is the true data generating process, Wald’s thresholds provide an upper limit to the effective *α* and *β* error rate, namely, $\alpha ^{\prime } \leq \nicefrac {1}{A}$ and $\beta ^{\prime } \leq B$ (see the online appendix at https://osf.io/5esbc/ for an outline of Wald’s proof). In certain situations, effective error rates can be substantially smaller than *α* and *β*, which means that Wald’s thresholds can overcontrol the error rates. The extent of overcontrol depends on the model specification. If the effect size postulated in the alternative model approaches zero, that is, if the null and alternative model get increasingly similar, the effective error rates approach the nominal error rates. If the alternative model postulates a large effect size, the SPRT overcontrols the error rates.

The reason for this imprecise error control is that the likelihood ratio often exceeds the thresholds rather than matching them exactly at the termination of the sequential process (Wald 1945, p. 132). This phenomenon of “overshooting” is more likely to happen when the models are dissimilar, because in these cases the likelihood ratio can be changed substantially by a single observation. In contrast, when the models are similar, each new observation causes only small changes in the likelihood ratio, which leads to less overshooting and a closer match in error rates. It is important to note that overshooting does not prohibit exact error control altogether. For dissimilar models, stopping thresholds that provide exact error control are narrower than Wald’s thresholds and can be found via numeric optimization methods (see Fig. 3 for an example with *δ*_0_ = 0 and *δ*_1_ = 0.5).

**Fig. 2:**
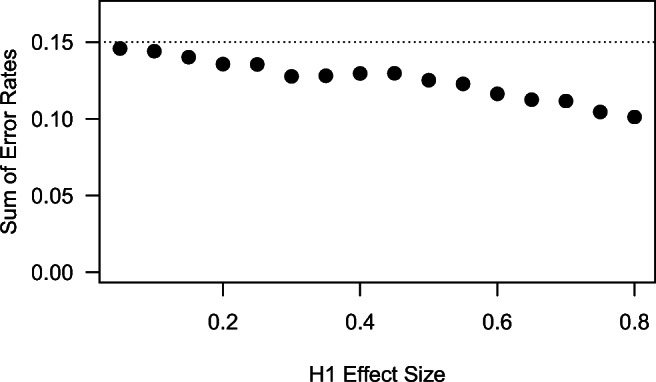
In the SPRT *t*-test with Wald’s thresholds, the sum of effective error rates approaches the sum of nominal error rates as the effect size postulated in the alternative model decreases. Depicted effective error rates are based on Monte Carlo simulations from the null and the alternative model with 10,000 iterations and Wald’s thresholds for nominal error rates of *α* = 0.05 and *β* = 0.1

Figure 2 demonstrates the dependence of error rates on the model specification in the SPRT with Wald’s thresholds for the *t*-test. The effective error rates (here, displayed as the sum of false-positive and false-negative errors) are based on Monte Carlo simulations from the null and the alternative model with 10,000 iterations and stopping according to Wald’s thresholds. Note that error control in the SPRT as displayed in the figure is conditional on the truthfulness of one of the two models, that is, the design no longer guarantees error control if a third model is the true data generating model. We will discuss this issue later in this paper.

#### SBFT: Symmetric and non-symmetric thresholds

In the SBFT, thresholds are often chosen to be symmetric around a Bayes factor of BF = 1 (Schönbrodt et al., 2017). For example, a researcher might choose to collect data until a Bayes factor larger than 10 or smaller than 1/10 is reached. This practice often signals that the researcher’s primary concern is strength of evidence rather than control of error rates. For example, a researcher might aim for a strength of evidence of 10, that is, the data should be at least 10 times more likely to have occurred under the selected model than under the competing model (Schönbrodt and Wagenmakers, 2018; Stefan et al., 2019).

Although it is common practice, there is no statistical reason for exclusively using symmetric thresholds in the SBFT. In fact, there are many reasons why a researcher might choose non-symmetric thresholds, for example to account for the fact that evidence accumulates more slowly for the null hypothesis than for the alternative hypothesis (Johnson & Rossell, 2010) or to incorporate utilities of hypotheses in the design (Good, 1983). As can be seen from Wald’s thresholds, non-symmetric thresholds are also beneficial if a researcher wants to control the rates of misleading evidence of their design. Adjusting the thresholds independently does not only allow the researcher to match the envisioned error rates of the design more closely, but also leads to lower average sample sizes (Stefan et al., 2019).

As in many other complex hypothesis testing scenarios (e.g., Green & MacLeod 2016), there is no analytical solution to obtain the error rates of an SBFT. However, optimal thresholds can be found in an iterative process using Bayes Factor Design Analysis (BFDA; Schönbrodt & Wagenmakers 2018), a simulation-based methodology that allows researchers to obtain the expected sample size and rates of misleading evidence of a sequential Bayesian design. In a BFDA, a large number of samples is generated from a population model representing the null or the alternative hypothesis, respectively. These samples are then analyzed using the sequential Bayesian design and the sample sizes and error rates of the design are tracked (Schönbrodt & Wagenmakers, 2018; Stefan et al., 2019). Optimal thresholds can be determined by defining an objective function on sample size and error rates and re-running the BFDA in an iterative process using optimization methods to find the thresholds that minimize the objective function. Notably, these optimal thresholds do not lead to an overcontrol of error rates because they are adjusted to the statistical models used in the test. We provide commented code for the simulation and optimization in the online appendix of this manuscript at https://osf.io/5esbc.

#### Same mechanism of error control

Both in the SPRT and in the SBFT, error rates can be controlled by adjusting the stopping thresholds of the design. Wald’s thresholds provide a computationally simple, analytic solution to the boundary optimization problem in the SPRT, but they lead to effective error rates that can be substantially smaller than the envisioned maximum error rates of the design. To obtain exact error control, simulation-based methods are necessary to determine optimal stopping thresholds in both sequential testing procedures.

**Fig. 3:**
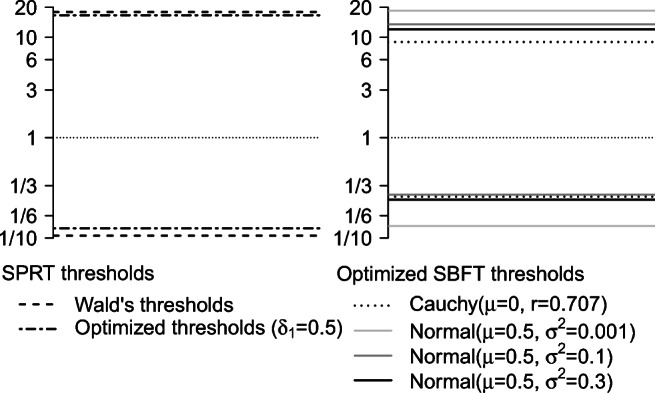
Stopping thresholds that provide exact error control in the SPRT and in the SBFT for $\alpha _{\max \limits } = 0.05$ and $\beta _{\max \limits } = 0.1$, as well as Wald’s thresholds. *Left panel*: Computationally optimized thresholds that provide exact error control in the SPRT are different from Wald’s thresholds. *Right panel*: Optimized thresholds for SBFT designs with different prior distributions (see legend). All thresholds are determined under the assumption of a population effect size of *δ* = 0.5

Figure 3 shows such computationally optimized thresholds for *α* = 0.05 and *β* = 0.1. Wald’s thresholds are wider than thresholds that provide exact error control, and therefore lead to an overcontrol of error rates. Additionally, it is evident from the figure that optimal thresholds depend on the model specification. The SPRT model and different Bayesian models clearly require different stopping thresholds to control the error rates at the same level.

#### Implications for sequential testing

Based on the previous sections, we can make three observations about threshold definition and error control in the SPRT and SBFT: (1) SPRT and SBFT control error rates through the same mechanism, namely threshold adjustment; (2) thresholds need to be defined through simulation methods in both tests if exact error control is desired; and (3) optimal thresholds depend on the model specification. Thus, if an SBFT with symmetric thresholds is compared to an SPRT with Wald’s thresholds, as has recently been proposed by Schnuerch and Erdfelder (2020), it is only natural that error control in the SBFT will be inferior to the SPRT because the thresholds in the SBFT were not adjusted to control error rates. In the same vein, it cannot be expected that the same stopping thresholds applied to two different designs will yield the same error rates. A difference in observed average sample sizes between the SPRT and SBFT can only be interpreted as a difference in test efficiency if the testing procedures are equated on at least one other dimension, that is, if they control error rates at the same level or if they yield the same amount of evidence. Researchers who wish to compare an SPRT and an SBF design therefore need to decide whether they want to impose the same stopping thresholds (i.e., the tests stop when the same amount of evidence is collected) or whether they want to compare two designs with the same error rates (i.e., differing stopping thresholds). We will present both types of comparison in the next sections.

## Which sequential testing procedure is more efficient?

In the previous sections, we showed that the SPRT and the SBFT are similar in many respects. Here, we show that differences in efficiency between the methods are gradual and depend on the exact model and design specification. Efficiency is important in all practical applications where time, money, or effort are proportional to the sample size required by a research design, or where the well-being of research subjects is affected by long testing procedures (Hunter & Hoff, 1967). In all sequential hypothesis testing procedures, sample size is a random variable that can vary between experiments. Therefore, we present average (expected) sample sizes for each procedure. As described earlier, we compare designs that either have the same error rates (i.e., optimized thresholds) or the same stopping thresholds (i.e., require the same amount of evidence for data collection to be stopped). This allows us to disentangle the effects of threshold definition on the expected sample size and on the error rates of the design. Since the difference between likelihood ratios in the SPRT and the Bayes factor in the SBFT depends on the definition of the prior distribution, we also use SBFTs with different prior distributions in our comparison.

### Efficiency under ideal conditions: A note on oracle priors

In the following two sections, we investigate the efficiency of the SPRT and SBFT under ideal conditions, where the true effect size matches the model expectations as closely as possible. This means that the population effect size is either equal to zero, i.e., the parameter postulated in the null hypothesis, or it matches the effect-size parameter (SPRT) or the mode of the prior distribution on effect size (SBFT) in the alternative model. We call this an *oracle prior* setup because it implies that researchers are able to make correct predictions about the population parameters. It should be noted that the SPRT naturally benefits from this setup, because the models compared in the SPRT are exact representations of the true population models. Due to the inherent uncertainty about parameter values, the efficiency of the SBFT can only approach the efficiency of the SPRT in these cases.

In reality, it is of course highly unlikely that the true data generating process matches the a-priori expectations of a researcher exactly (Wald, 1945; Box, 1976). However, it is still relevant to compare models under idealized circumstances because they describe a reliable best-case scenario with minimal expected sample sizes and effective error control. Any model misspecification distorts the stated properties of the hypothesis tests, most prominently, the desired error rates of the design. We will analyze the behavior of the two hypothesis testing methods under the condition of model misspecification after the investigation of the idealized scenario.

### Efficiency of one-sided tests

In the following, we will use Monte Carlo simulations to compare the efficiency of the one-sided SPRT *t*-test and the one-sided sequential Bayesian *t*-test in an oracle-prior setup. As outlined before, we compare tests that are either matched in terms of error rates or in terms of stopping thresholds. We use true effect sizes of *δ* = 0.2, *δ* = 0.5, and *δ* = 0.8 under the alternative hypothesis, and a true effect size of *δ* = 0 under the null hypothesis. We decided to use these effect sizes because they cover the range of typical effect sizes in psychological studies (Wetzels et al., 2011) and have been used in earlier studies comparing the SPRT and SBFT (Schnuerch & Erdfelder, 2020). In the SBFT, we use four different prior distributions on effect size under the alternative model for each comparison: A default zero-centered Cauchy distribution with scale $r = \sqrt {2}/2$ and informed normal distributions that are centered on the population effect size and have variances of *σ*^2^ = 0.3, *σ*^2^ = 0.1, or *σ*^2^ = 0.001. We decided to use three informed priors with increasingly smaller variances to be able to demonstrate what happens if the informed prior approaches a point prior. In the SPRT, the effect size postulated in the alternative model is always equal to the simulated population effect size. In the SBFT, the simulated population effect size is equal to the mean of the informed prior. Analysis code and tables can be found in our online appendix (https://osf.io/5esbc/).

#### Same thresholds

**Fig. 4:**
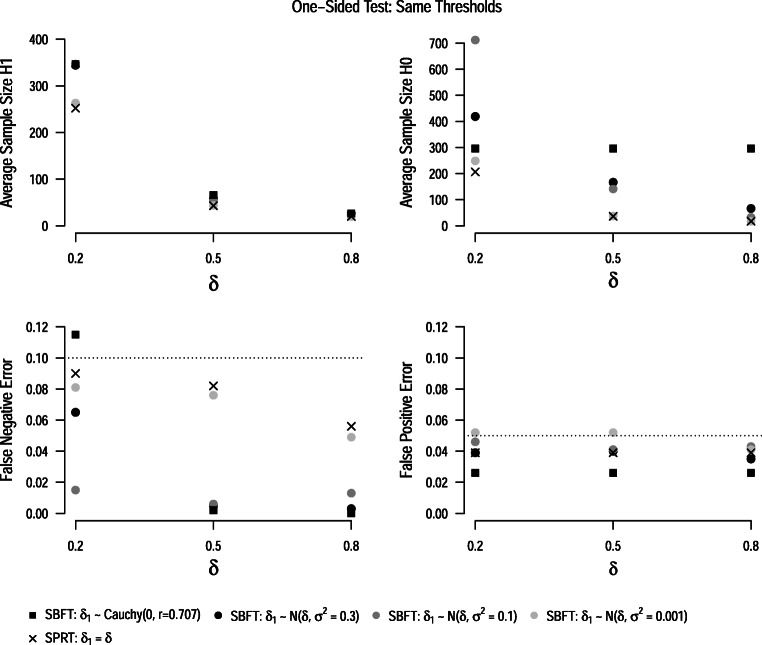
Average sample sizes and error rates for the one-sided SPRT *t*-test (*crosses*) and sequential Bayesian *t*-test (*circles*, *squares*) with Wald’s thresholds. All results are based on the assumption of an oracle prior, i.e., that the true effect size is either zero or the parameter *δ*_1_ specified in the alternative model of the SPRT. *Dotted lines* show the maximum false-positive and false-negative error rates

In the following, we apply Wald’s thresholds for maximum error rates of *α* = 0.05 and *β* = 0.1 to both sequential hypothesis tests. We decided to use Wald’s thresholds because they are commonly recommended for the SPRT (Wald, 1945; Schnuerch & Erdfelder, 2020), and they allow researchers to control error rates (although issues of overcontrol exist, as discussed earlier). Figure 4 displays the results of these analyses. The upper panels display the average sample sizes under the null and alternative hypothesis. In all cases, the SPRT has the lowest average sample size. This can be expected since the true data generating process exactly matches the models postulated in the SPRT. The average sample sizes in the SBFT approach the SPRT as the prior distribution becomes narrower around the population parameter. The wide, zero-centered default prior distribution typically leads to the highest average sample sizes. This result corroborates earlier simulation results by Schnuerch and Erdfelder (2020), and can be explained by the (dis)similarity between the Bayes factor and likelihood ratio discussed earlier (see also Fig. 1). Another interesting aspect visible in the figure is that average sample sizes in SPRT and SBFT are more similar if the (postulated) population effect size under the alternative hypothesis is large. In this case, both tests can capitalize on faster rates of evidence accumulation and typically stop data collection after a very small number of observations. The lower panels of Fig. 4 show the false-positive and false-negative error rates of the designs when Wald’s thresholds are imposed on all tests. It becomes clear that Wald’s thresholds do not control error rates for all configurations of the SBFT; however, the deviations from the maximum false-positive and false-negative error rates are typically minor. In fact, Wald’s thresholds also overcontrol error rates for most SBFT settings.

#### Same error rate

In the following, we optimized thresholds in the SBFT to yield the same effective error rates as the SPRT with Wald’s thresholds, i.e., a false-positive error rate of *α* = 0.039, and false-negative error rates of *β* = 0.090,*β* = 0.082, and *β* = 0.052 for population effect sizes of *δ* = 0.2,*δ* = 0.5, and *δ* = 0.8, respectively (see crosses in Fig. 4).

Finding the optimal thresholds that can provide exact error control with minimal sample sizes in the SPRT or SBFT requires an efficient optimization procedure. At the heart of the optimization procedure is an objective function that takes in the two threshold values, and computes a summary statistic (e.g., the mean) of the expected sample sizes under the null and alternative model, while penalizing for error rates that exceed the desired levels. This objective function can be minimized using a standard multidimensional optimization algorithm. Here, we used a differential evolution algorithm as implemented in the R package *NMOF* (Schumann, 2019). Differential evolution is a derivative-free stochastic optimization algorithm that was developed to be applied to continuous-valued problems (Engelbrecht, 2007). The calculation of error rates and expected sample sizes requires a large number of Monte Carlo simulations of the sequential process under each of the two competing models. As the data generating models remain constant regardless of the thresholds, it is possible to draw the Monte Carlo samples once at the beginning of the optimization for each hypothesis, and limit the objective function to “cutting off” the sampled trajectories based on the proposed thresholds. This reduces computing time for the optimization from several hours to a few seconds, since Bayes factors or likelihood ratios only have to be computed on the original sample. The optimization procedure yields evidence thresholds for the SBFT that are model-specific and lead to an exact matching to the effective error rates of the SPRT while keeping the average sample sizes at a minimum. We provide commented sample code with customizable functions for the threshold optimization procedure in our online appendix (https://osf.io/5esbc/).

**Fig. 5:**
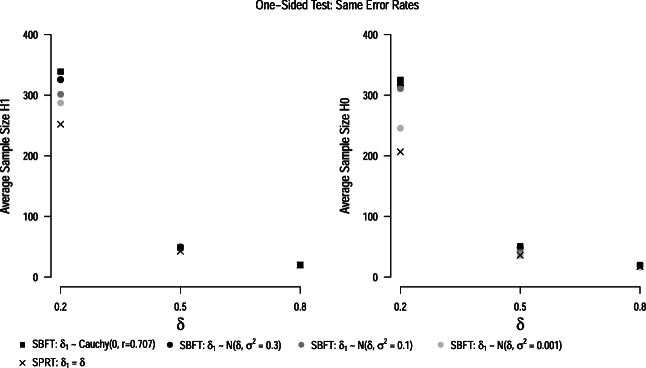
Average sample sizes for the one-sided SPRT *t*-test (*crosses*) and sequential Bayesian *t*-test (*circles*, *squares*) when thresholds are optimized so that the tests yield the same error rates. All results are based on the assumption of an oracle prior, i.e., that the true effect size is either zero or the parameter *δ*_1_ specified in the alternative model of the SPRT

As can be seen from Fig. 5, the SPRT yielded lower average sample sizes than the SBFT when thresholds are optimized such that the tests have the same error rates. However, the differences in average sample size are not as pronounced as when the same thresholds are applied to both testing procedures. A reason for this is that the optimized thresholds in the SBFT are typically narrower than the thresholds yielding the same error rates in the SPRT, as can be seen from Fig. 3.

### Efficiency of two-sided tests

As we discussed earlier, the sidedness of a sequential test influences the models involved in the test. Therefore, it is important to consider both one-sided and two-sided tests when comparing the efficiency of the SBFT and of the SPRT. In the previous sections, we compared the two procedures for one-sided tests. To provide a comprehensive comparison, we repeated all simulations with two-sided tests. Unless otherwise mentioned, the comparison setup is identical to the one-sided analyses. Informed prior distributions in the SBFT are unimodal and peak at the simulated population effect size. Analysis code and tables can be found in our online appendix (https://osf.io/5esbc/).

#### Same thresholds

**Fig. 6:**
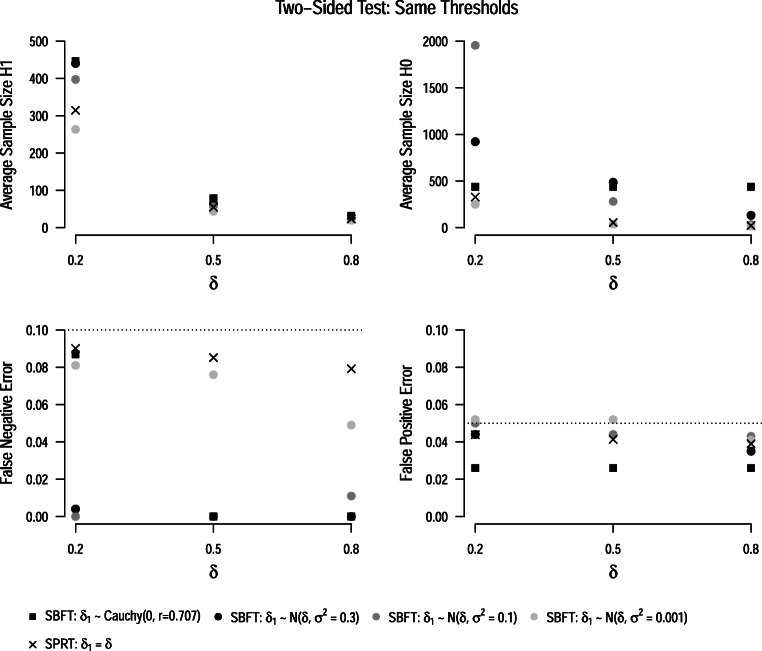
Average sample sizes and error rates for the two-sided SPRT *t*-test (*crosses*) and sequential Bayesian *t*-test (*circles*, *squares*) with Wald’s thresholds. All results are based on the assumption of an oracle prior, i.e., that the true effect size is either zero or the parameter *δ*_1_ specified in the alternative model of the SPRT. *Dotted lines* show the maximum false-positive and false-negative error rates

Figure 6 displays the average sample sizes under the null and under the alternative model for the two-sided test when Wald’s bounds are applied to both procedures. Interestingly, we can see that the SPRT *t*-test is no longer more efficient than the sequential Bayesian *t*-test. Across all conditions, the sequential Bayesian design with an extremely narrow prior distribution (*σ*^2^ = 0.001) has lower average sample sizes. How can this be explained? As we outlined earlier, the SPRT model is defined in absolute terms, that is, the squared *t*-value is entered into the likelihood. However, raw values occur on the real line, which means that the alternative hypothesis in the test effectively places a point prior on both + Δ and −Δ. In contrast, one-sided and two-sided models in the SBFT differ due to the truncation of the prior distribution. While the prior distribution covers the whole range of values in the two-sided case, it is truncated at *δ* = 0 in the one-sided case. However, for highly informed prior distributions, the truncation does not change the probability densities of the distribution much because the full distribution assigned only little prior mass to values in the truncated area. Therefore, for (highly) informed prior distributions, the two-sided Bayesian *t*-test is more similar to the one-sided Bayesian *t*-test than to the two-sided SPRT.

#### Same error rates

**Fig. 7:**
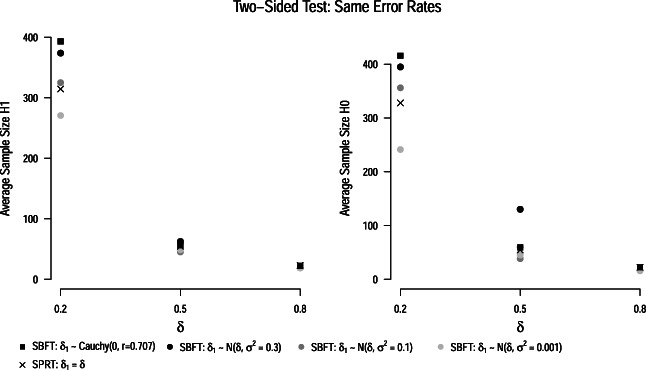
Average sample sizes for the one-sided SPRT *t*-test (*crosses*) and sequential Bayesian *t*-test (*circles*, *squares*) when thresholds are optimized so that the tests yield the same error rates. All results are based on the assumption of an oracle prior, i.e., that the true effect size is either zero or the parameter *δ*_1_ specified in the alternative model of the SPRT

When error rates are controlled through optimized stopping thresholds, the same pattern emerges (see Fig. 7). The two-sided SBFT with an extremely narrow prior distribution yields lower average sample sizes than the two-sided SPRT. The default Cauchy prior typically yields the highest average sample sizes.

The question remains whether any of the most efficient models in the two-sided case can be justified from a model building perspective. Neither a “double” point prior on an effect size and its additive inverse, nor a spiked prior on an effect size that allows for effect sizes of a different sign seems to be an intuitive choice in most application scenarios. We will discuss the question of theory-based model specification later in this manuscript.

### Robustness against model misspecification

In the SPRT and sequential Bayesian tests, expectations about the population effect size determine the model specification. The previous sections assumed that these expectations were true, that is, that either the null or the alternative model of the SPRT were the data generating process. However, in real-life applications, it is fair to assume that this idealized scenario rarely if ever applies (Wald, 1945; Box, 1976). The ensuing model misspecification distorts the properties of the hypothesis test.

In the following, we explore how model misspecification influences the average sample sizes and the test decisions in the SPRT and in the sequential Bayesian *t*-test. To ensure continuity to the previous sections, we compare the SPRT and the SBFT with the same settings for expected effect sizes and prior distributions. In our simulations of model misspecification, we allowed for population effect sizes under the alternative model between *δ* = 0.1 and *δ* = 1.0. This implies that the (most likely) effect size *δ*_1_ specified in the alternative model can both over- and underestimate the true effect size. We compare directional designs that yield the same error rates if either of the specified models is true. In the SPRT, the error rates were controlled using Wald’s thresholds. Error rates in the SBFT were matched to the effective error rates in the SPRT using the optimization procedure described earlier in this paper.

#### False-negative results

**Fig. 8:**
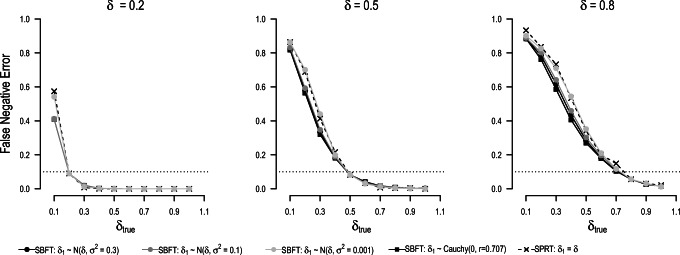
False-negative error rates for the SPRT and sequential Bayesian *t*-test when the true effect size *δ*_true_ does not match the effect size postulated in the null or alternative model (*δ*_0_ = 0 and *δ*_1_ = *δ*, respectively)

Figure 8 depicts the rate of test decisions in favor of the null hypothesis. Given that the simulated true population effect size is always larger than zero, this rate can be considered the false-negative error rate of the design. If the true effect size is smaller than expected, the false-negative error rate of the design exceeds the nominal error rate. The results show that throughout all conditions, false-negative error rates in the SPRT are higher than in the SBFT. This demonstrates that the SBFT is a more conservative testing procedure that is more robust against model misspecification than the SPRT in terms of false-negative error rates. The reason for this is that the Bayesian test spreads probability across an effect size range, such that multiple effect sizes can be considered to be in accord with the model. As in the previous analyses, the properties of the SBFT are more similar to the SPRT if narrow prior distributions are chosen.

#### Average sample sizes

**Fig. 9:**
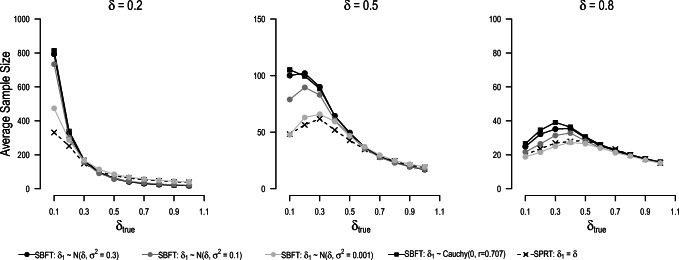
Sample sizes for the SPRT and sequential Bayesian *t*-test when the true effect size *δ*_true_ does not match the effect size postulated in the null or alternative model (*δ*_0_ = 0 and *δ*_1_ = *δ*, respectively)

Figure 9 depicts the average sample sizes under different degrees of model misspecification. For all conditions, average sample sizes increase if the true population effect size is smaller than expected. However, if the population effect size approaches zero, the average sample sizes decrease again in many conditions. As can be seen from the increasing false-negative error rates in Fig. 8, this can be explained by early termination in favor of the null model. If the true effect size is larger than expected, average sample sizes decrease compared to the oracle prior scenario. Interestingly, this decrease is particularly strong for the SBFT with wide prior distributions in the alternative model. If the effect size is sufficiently large, the average sample size for the SBFT can even be smaller than for the SPRT (see, e.g., the left panel of Fig. 9 for large *δ*_true_). The reason is that wide prior distributions assign a higher plausibility to large effect sizes, which means that these models make better predictions if these large effect sizes materialize.

### Implications for sequential testing

Our efficiency comparisons showed that the SPRT requires substantially smaller average sample sizes than the SBFT under ideal conditions, that is, when the true population effect size equals the effect size proposed in the alternative model of the SPRT. However, this increased efficiency comes at the cost of lower robustness against misspecifications. If the population effect size is smaller than the effect size proposed in the alternative model of the SPRT, error rates in the SPRT are higher than in the SBFT. If the population effect size is larger than expected, the SPRT eventually has larger average sample sizes than the SBFT. It can therefore be concluded that the SPRT benefits substantially from an oracle prior simulation setup. Generally, the design properties of the SBFT approach the SPRT when increasingly narrow prior distributions under the alternative model are used. This means that SBFTs with highly informed prior distributions are more efficient in terms of average sample sizes, but also less robust against model misspecifications than SBFTs with wide prior distributions. Taken together, our results indicate that differences in the SPRT and the SBFT are contingent on the specifics of model formulation as well as on the data generating process at the population level. Researchers planning a sequential hypothesis test should be aware that the model and threshold specification dynamically influence the properties of the planned design. In the next section, we provide guidance on how to navigate these design decisions.

## How to choose a sequential testing procedure in real-world applications

In this final section, we address the question how researchers can decide on a model specification for real-world applications. We provide several guiding principles and investigate pragmatic research strategies that have been proposed with regard to the SPRT and SBFT.

### Uncertainty specification

The SBFT assumes uncertainty about parameter values under the alternative hypothesis (Schönbrodt et al., 2017) while this is not the case for the SPRT (Wald, 1945; Hajnal, 1961). Therefore, one important consideration in the choice of the sequential hypothesis testing framework is whether uncertainty about parameter values should be quantified.

From a theoretical standpoint, the question arises which situations warrant the specification of uncertainty. Arguably, only a very limited number of research contexts provides researchers with sufficient background knowledge to confidently define a single specific effect size under each of the competing models. For example, researchers in physics might wonder whether a physical constant takes one value or another, or mechanical engineers might be interested in the question whether a certain object was built with either of two materials. In the social sciences, however, it is difficult to find examples where a theory provides researchers with an unequivocal single effect size. In fact, in Bayesian statistics, there has even been a longstanding debate whether research contexts typically provide sufficient reliable prior information for the specification of informed prior distributions (Goldstein, 2006; Fienberg, 2006). A substantial number of Bayesian statisticians advocate for the use of wide default prior distributions because they reflect a high amount of uncertainty, such as the Cauchy distribution used here (Savage, 1954; Consonni et al., 2018). Moreover, in the Bayesian statistical framework, learning about parameter values from the data becomes impossible when a point prior is used because the prior distribution cannot be meaningfully updated to a posterior distribution (Etz & Vandekerckhove, 2018). Thus, from a Bayesian perspective, formulating a point prior on parameter values in the alternative model in the face of uncertainty seems rash or even reckless. However, proponents of the SPRT argue that it is not necessary to view the specified parameters as a researcher’s best guess for the underlying population effect (Schnuerch & Erdfelder, 2020). Instead, they argue that the parameter values can be interpreted as the smallest effect size researchers are interested in detecting. This view stems from approaches to power analysis where a smallest effect size of interest (SESOI) or the lower threshold of a confidence interval is used to plan for sufficiently large sample sizes (Lakens & Evers, 2014; Perugini et al., 2014). However, this leads to a model that can be no longer interpreted as a manifestation of theory, as we will discuss below.

### Model predictions

Traditionally, statistical models are interpreted as mathematical manifestations of theories (Vanpaemel, 2010). From this perspective, one of the key aspects of a model is its generative property, that is, its ability to make realistic predictions that are in accord with a theory (Gelman et al., 2017; Vanpaemel, 2010). If a researcher acknowledges the null and alternative model as justifiable manifestations of plausible theories, the results of the test can be directly interpreted as a test of theory.

In the SPRT, researchers following a theory-driven model specification approach would engage in a “Best Guess” heuristic when determining the effect size for the alternative model. This means that they would specify the parameter based on their beliefs about the most likely parameter values under a given theory (Dienes, 2019). Note that by definition a SESOI specification of the effect size parameter does not represent a researcher’s best guess, but a lower bound on what the researcher deems realistic or interesting (e.g., Button et al., 2015; Simonsohn 2015; Perugini et al., 2014; Lakens et al., 2018). Thus, the SESOI specification results in an effect size that is likely to underestimate the true population effect size and can lead to biased model predictions. From a purely frequentist point of view, this may be viewed as unproblematic since the primary focus is on error control, and model predictions are only secondary.

In the SBFT, informed prior distributions allow models to make theoretically meaningful predictions (Lee & Wagenmakers, 2013). Informed prior distributions can be formulated based on theoretical considerations, previous literature, or beliefs of substantial experts (Lee & Vanpaemel, 2017; Verhagen & Wagenmakers, 2014; Stefan et al., 2020). If there is a high degree of uncertainty about the effect size, default prior distributions, such as the central Cauchy, can also yield models with realistic predictions that reflect researchers’ theory-based beliefs. However, researchers arguably often have more pre-data knowledge about an effect size than the vague predictions of default prior distributions suggest (Dienes, 2019). Therefore, the use of default prior distributions is in practice rarely driven by substantive theories and often results in unrealistic model predictions (Stefan et al., 2020).

### Design planning vs. model inference

An interesting aspect about the SPRT and SBFT is that design planning and model inference are not independent of each other. As we showed before, optimal decision thresholds that control error rates are dependent on the model specification, and average sample sizes depend on the combination of models, thresholds, and true population effect size. Researchers eager to save resources might be tempted to start planning a sequential hypothesis test by examining the average sample sizes of different sequential hypothesis testing setups, and selecting the one that promises conclusive results with minimal sample sizes. However, the test yielding the lowest average sample sizes under ideal conditions is not necessarily an appropriate test for a specific research scenario. For example, an SPRT postulating an effect size of *δ* = 1 under the alternative model may stop after few observations, but it might not compare models that are realistic for social science where effect sizes are typically smaller (Wetzels et al., 2011). More generally, restricting prior distributions on effect size under the alternative model to a small range of values can result in considerable efficiency gains, but it can also lead to more frequent model rejections and can therefore be regarded as a risky choice in terms of design planning. Due to dependencies like the ones outlaid, researchers planning an SPRT or SBFT always need to consider the interplay between efficiency and model inference in the design planning process. However, it might be useful to remember at this point that even the least efficient sequential design is typically more efficient than the most efficient design with fixed sample sizes. Therefore, researchers may benefit from considering the aspect of design efficiency in a broader context.

One sequential testing configuration that stands out in terms of design planning is the SBFT with default prior distributions. Models with default prior distributions signal a high a-priori uncertainty about the effect size (Consonni et al., 2018). Unlike informed or point priors, default priors do not place much prior mass on any particular parameter values. Therefore, any parameter value can reasonably be assumed to be the true effect size during design planning (this parameter value is also known as the “design prior”; Schönbrodt & Wagenmakers 2018; Stefan et al., 2019). The thresholds of the default SBFT can be constructed with regard to this effect size, such that error rates of the design are controlled if the true population effect size is equal to or larger than the specified design prior (see for examples Figs. 5 and 8). In practice, the design prior could for example be a best guess or a SESOI, but theoretically, the error rates of the design can be controlled with regard to any postulated effect size, without having to assign a high weight to this effect size in the model itself (Schönbrodt & Wagenmakers, 2018; Stefan et al., 2019). Therefore, for models with default prior distributions, design planning can be disentangled from model specification. Although this is technically possible for models with informed prior distributions, it is only theoretically meaningful for default prior distributions.

### A Comparison of pragmatic research strategies

In the previous sections, we mentioned several pragmatic research strategies that have been proposed with regard to sequential hypothesis testing. Researchers can decide to use an SPRT based on a best guess or a smallest effect size of interest, or they can opt for an SBFT with informed or default prior distributions. Each of the proposed strategies has advantages and disadvantages that might manifest themselves to a different extent in different research contexts. Table 1 summarizes these properties of the research strategies that were described in the previous sections. Researchers deciding for a sequential testing procedure must carefully weigh the advantages and disadvantages in the context of their application domain. For example, researchers in a field with high uncertainty about effect sizes might find uncertainty quantification more important than researchers who work in fields with theories that make precise predictions. It is important to note that the “informed SBFT” strategy in fact incorporates a wide variety of prior specifications that can morph into all of the other categories (as we explained in the section on evidence monitoring earlier in this manuscript). Therefore, the strategies listed in Table 1 should not be understood as distinct approaches, but as archetypal examples on a continuous dimension. It is also important to mention that the list in Table 1 assumes that a researcher has already decided to use a sequential hypothesis testing procedure (as opposed to a fixed sample design). Therefore, the items are limited to relative advantages and disadvantages *within* the sequential hypothesis testing framework.

**Table 1:** Advantages and disadvantages of pragmatic research strategies

Research strategy	Advantages (✔) and Disadvantages (✖)
SESOI SPRT	✔ Small average sample sizes compared to SBFT*
	✔ Robust error control
	✖ Unrealistic model predictions
	✖ Uncertainty quantification impossible
Best-Guess SPRT	✔ Small average sample sizes compared to SBFT*
	✔ Theoretically meaningful model predictions
	✖ Highly susceptible to model misspecification
	✖ Uncertainty quantification impossible
Informed SBFT	✔ Theoretically meaningful predictions
	✔ Smaller average sample sizes than default SBFT*
	✖ Larger average sample sizes than SPRT*
	✖ More susceptible to model misspecification than default SBFT
Default SBFT	✔ Can be specified without prior knowledge about effect size
	✔ Independence of design planning and model specification
	✖ Likely to yield unrealistic model predictions
	✖ High average sample sizes compared to all other methods*

*Note:* SESOI SPRT: SPRT with *δ*_1_ = Smallest Effect Size of Interest; Best-Guess SPRT: SPRT with *δ*_1_ = best guess / constant predicted by theory; Informed SBFT: SBFT with informed prior distribution; Default SBFT: SBFT with default prior distribution; * in an ideal (oracle prior) scenario

## Conclusions

Sequential hypothesis testing procedures constitute a powerful tool to achieve experimental efficiency (Wald & Wolfowitz, 1948). In this manuscript, we compared two sequential hypothesis testing procedures that have been proposed for the use in psychological research, the sequential probability ratio test (SPRT; Wald 1945; Hajnal 1961) and the Sequential Bayes Factor Test (SBFT; Schönbrodt et al., 2017). We showed that recent efforts to compare the two designs have exaggerated the differences between the two approaches and that, philosophical differences notwithstanding, the choice between the two methods can be regarded as a continuous choice within a unified framework rather than a dichotomous decision. We demonstrated that differences in efficiency between the methods are gradual, and discussed how the desideratum of efficiency needs to be weighed against other desiderata (e.g., robustness against model misspecification) when choosing a sequential testing design.

In this paper, our focus has been to lay out similarities between the SPRT and SBFT. Nevertheless, it should be noted that the two hypothesis testing procedures are associated with different philosophies of statistical testing. While the SPRT has been developed to optimize the balance between error control and expected sample size (Wald, 1945; Wald & Wolfowitz, 1948), the SBFT has been proposed with a focus on Bayesian evidence strength (Schönbrodt et al., 2017). This raises the question whether, from a theoretical point of view, a sequential design that takes both evidence strength *and* error rates into account should be classified as an SBFT, an SPRT, or a new hybrid methodology. For example, a researcher might initially define stopping thresholds based on error rate considerations, but then widen the thresholds if they do not deem the resulting evidence compelling. Or a researcher might decide to monitor a likelihood ratio but define stopping thresholds based on evidence strength. Indeed, hybrid forms of sequential hypothesis tests can already be found in the early literature on sequential testing, among others in Wald’s own work regarding SPRTs for composite hypotheses (Wald 1945, p. 181 ff.). Sequential tests for composite hypotheses have also been proposed more recently in the context of ability testing with IRT (Thompson & Ro, 2007). Here, we take the stance that it is not necessary to proclaim a new hybrid methodology for each of these applications. In our opinion, design decisions on the continuum between SPRT and SBFT can reflect different philosophies of statistical testing as well as different priorities with regard to the operating characteristics of the test.

Both sequential hypothesis testing methods discussed in this manuscript have been shown to be substantially more efficient than comparable tests with fixed sample sizes (Schnuerch & Erdfelder, 2020; Wald & Wolfowitz, 1948; Schönbrodt et al., 2017). They can also be considered superior to traditional frequentist sequential hypothesis testing procedures such as group sequential designs (Pocock, 1977; O’Brien & Fleming, 1979) because they do not require a strict schedule for interim analyses and do not lose statistical power with an increasing number of interim analyses. Thus, SPRT and SBFT can yield substantial efficiency gains for experiments in scientific fields that decide to adopt them.

Many results in our paper rely on finding a sequential design with pre-specified error rates by optimizing the stopping thresholds. In the literature, many examples for simulation-based power analyses for sequential designs with fixed thresholds can be found (e.g., Schönbrodt & Wagenmakers 2018; Stefan et al., 2019; Schnuerch & Erdfelder 2020). However, to the best of our knowledge, optimizing thresholds using numeric optimization algorithms to obtain certain design characteristics is a novel idea. We believe that the procedure will be most relevant to researchers using the SBFT, since Wald’s thresholds are not guaranteed to provide effective error control for Bayesian sequential designs,^3^ and optimal thresholds often differ substantially from Wald’s thresholds. In the SPRT, optimizing thresholds might only lead to a small absolute reduction in average sample sizes, since Wald’s thresholds deviate most from the optimal thresholds for large effect sizes, where expected sample sizes are already low. However, when each observation is costly or data collection is time consuming, these small efficiency gains on the scale of average sample sizes may be equivalent to large reductions in the study cost or study duration. Therefore, we believe that researchers conducting an SPRT may also be interested in threshold optimization. In the online appendix of this paper, we provide commented R code for the threshold optimization procedure in the SBFT and SPRT, which we hope will be easy to use and adapt for researchers in practice.

The SPRT and SBFT are both guaranteed to stop at a finite sample size (Wald, 1945; Ly et al., 2016). However, a limitation of the procedures is that they are not guaranteed to end at a sample size that is practically feasible. This means that researchers might be forced to stop sampling early due to a lack of resources. In this case, no test decision can be made, but the monitored outcome of the tests, that is, the Bayes factor or likelihood ratio, can still be interpreted as evidence strength in favor of the models (Rouder, 2014). Existing adaptations of the procedures, such as the maxSBF design (Schönbrodt et al., 2017) or the MSPRT (Pramanik et al., 2020), have attempted to resolve this issue. Depending on the maximum sample size defined for the procedure, these methods can yield very similar results to the SPRT or the SBFT described in this manuscript (Schönbrodt and Wagenmakers, 2018; Pramanik et al., 2020).

In sum, sequential hypothesis tests provide an important addition to a researcher’s methodological toolbox and can substantially increase the efficiency of research designs. Similar to other statistical analysis methods, it is crucial that researchers employing these methods are familiar with their theoretical assumptions and practical implications. Here, we investigated the theoretical underpinnings of two sequential methods, the SPRT and the SBFT, and discussed several guiding principles for their application. We hope that this provides researchers with the necessary knowledge to find the adequate sequential hypothesis testing strategy for their application domain.

## Open Practices Statement

Associated materials can be found at https://osf.io/5esbc/. Reproducible analysis code is available at https://osf.io/5esbc/. Reported analyses were not preregistered.

## Footnotes

1 Throughout the manuscript we assume that a test is conducted after each datapoint. However, sequential tests also allow for larger sets of data to be collected at each step. This will influence the sample sizes and error rates of the design.

2 Note that SPRT *t*-tests are technically testing composite hypotheses, as a weight function (i.e., prior) of 1/*V* *a**r* on the variance is implied under both models (Cox, 1952). Therefore, they are not optimal tests in the sense of Wald and Wolfowitz (1948). However, in all other aspects they are equivalent to Wald’s SPRT (Wald, 1945, p. 181f; Cox, 1952), which makes the distinction of limited practical importance (Cox, 1952). In fact, Lai (1981) showed that Wald and Wolfowitz’s proof may also hold asymptotically for SPRT *t* tests.

3 Schnuerch et al., (2021) make a point that the weighted average of all type II error rates based on the effect sizes specified in the prior distribution under ${\mathscr{M}}_{1}$ will be smaller than the maximum error rate. However, this does not imply that the effective error rate is smaller than the specified error rate for any specific effect size.

## References

[CR1] Armitage P, McPherson CK, Rowe BC (1969). Repeated significance tests on accumulating data. Journal of the Royal Statistical Society. Series A (General).

[CR2] Berger J (2006). The case for objective Bayesian analysis. Bayesian Analysis.

[CR3] Bogacz R, Brown E, Moehlis J, Holmes P, Cohen JD (2006). The physics of optimal decision making: a formal analysis of models of performance in two-alternative forced choice tasks. Psychological Review.

[CR4] Box GEP (1976). Science and statistics. Journal of the American Statistical Association.

[CR5] Brereton RG (2015). The F distribution and its relationship to the chi squared and t distributions: The F distribution. Journal of Chemometrics.

[CR6] Button KS, Kounali D, Thomas L, Wiles NJ, Peters TJ, Welton NJ (2015). Minimal clinically important difference on the Beck Depression Inventory - II according to the patient’s perspective. Psychological Medicine.

[CR7] Consonni G, Fouskakis D, Liseo B, Ntzoufras I (2018). Prior distributions for objective Bayesian analysis. Bayesian Analysis.

[CR8] Cox DR (1952). Sequential tests for composite hypotheses. Mathematical Proceedings of the Cambridge Philosophical Society.

[CR9] Dienes Z (2019). How do I know what my theory predicts?. Advances in Methods and Practices in Psychological Science.

[CR10] Dupont WD, Plummer WD (1990). Power and sample size calculations: a review and computer program. Controlled Clinical Trials.

[CR11] Eggen T (2011). Computerized classification testing with the Rasch model. Educational Research and Evaluation.

[CR12] Eggen T, Straetmans G (2000). Computerized adaptive testing for classifying examinees into three categories. Educational and Psychological Measurement.

[CR13] Engelbrecht, A.P. (2007). Differential evolution. In A.P. Engelbrecht (Ed.) *Computational intelligence: An introduction*. (2nd edn.) (pp. 237–260). Chichester: Wiley.

[CR14] Etz A, Haaf JM, Rouder JN, Vandekerckhove J (2018). Bayesian inference and testing any hypothesis you can specify. Advances in Methods and Practices in Psychological Science.

[CR15] Etz A, Vandekerckhove J (2018). Introduction to Bayesian inference for psychology. Psychonomic Bulletin Review.

[CR16] Ferguson, R.L. (1969). The development, implementation, and evaluation of a computer-assisted branched test for a program of individually prescribed instruction. Unpublished doctoral dissertation.

[CR17] Fienberg SE (2006). Does it make sense to be an “objective Bayesian”? (comment on articles by Berger and by Goldstein). Bayesian Analysis.

[CR18] Finkelman M (2008). On using stochastic curtailment to shorten the SPRT in sequential mastery testing. Journal of Educational and Behavioral Statistics.

[CR19] Gelman A, Simpson D, Betancourt M (2017). The prior can often only be understood in the context of the likelihood. Entropy.

[CR20] Goldstein M (2006). Subjective Bayesian analysis: Principles and practice. Bayesian Analysis.

[CR21] Good IJ (1983). Good thinking: The foundations of probability and its applications.

[CR22] Green P, MacLeod CJ (2016). SIMR: An R Package for power analysis of generalized linear mixed models by simulation. Methods in Ecology and Evolution.

[CR23] Griffith T, Baker S-A, Lepora NF (2021). The statistics of optimal decision making: Exploring the relationship between signal detection theory and sequential analysis. Journal of Mathematical Psychology.

[CR24] Gronau QF, Ly A, Wagenmakers E-J (2020). Informed Bayesian t-Tests. The American Statistician.

[CR25] Hajnal J (1961). A two-sample sequential t-test. Biometrika.

[CR26] Hunter WG, Hoff M (1967). Planning experiments to increase research efficiency. Industrial and Engineering Chemistry.

[CR27] Jeffreys H (1961). Theory of probability (Third ed.).

[CR28] John LK, Loewenstein G, Prelec D (2012). Measuring the prevalence of questionable research practices with incentives for truth telling. Psychological Science.

[CR29] Johnson VE, Rossell D (2010). On the use of non-local prior densities in Bayesian hypothesis tests. Journal of the Royal Statistical Society: Series B (Statistical Methodology).

[CR30] Kass RE, Raftery AE (1995). Bayes factors. Journal of the American Statistical Association.

[CR31] Lai TL (1981). Asymptotic optimality of invariant sequential probability ratio tests. The Annals of Statistics.

[CR32] Lakens D, Evers ERK (2014). Sailing from the seas of chaos into the corridor of stability: Practical recommendations to increase the informational value of studies. Perspectives on Psychological Science.

[CR33] Lakens D, Scheel AM, Isager PM (2018). Equivalence testing for psychological research: A tutorial. Advances in Methods and Practices in Psychological Science.

[CR34] Lee MD, Vanpaemel W (2017). Determining informative priors for cognitive models. Psychonomic Bulletin & Review.

[CR35] Lee MD, Wagenmakers E (2013). Bayesian Cognitive Modeling: A Practical Course.

[CR36] Li X, Liu J, Ying Z (2014). Generalized sequential probability ratio test for separate families of hypotheses. Sequential Analysis.

[CR37] Lin, C.-j., & Spray, J. (2000). Effects of item-selection criteria on classification testing with the sequential probability ratio test. ACT Research Report Series, P.

[CR38] Lindley D (2004). That wretched prior. Significance.

[CR39] Luce DR (1986). Response Times: Their Role in Inferring Elementary Mental Organization.

[CR40] Ly A, Verhagen J, Wagenmakers E-J (2016). Harold Jeffrey’s default Bayes factor hypothesis tests: Explanation, extension, and application in psychology. Journal of Mathematical Psychology.

[CR41] Mani N, Schreiner MS, Brase J, Köhler K, Strassen K, Postin D (2020). Sequential bayes Factor designs in developmental research: Studies on early word learning. Developmental Science.

[CR42] Milosavljevic M, Malmaud J, Huth A, Koch C, Rangel A (2010). The drift diffusion model can account for the accuracy and reaction time of value-based choices under high and low time pressure. Judgment and Decision Making.

[CR43] Morey, R., & Rouder, J.N. (2018). BayesFactor: Computation of Bayes factors for common designs. https://cran.r-project.org/web/packages/BayesFactor/index.html

[CR44] Myung JI, Pitt MA (2009). Optimal experimental design for model discrimination. Psychological Review.

[CR45] Neyman J, Pearson ES (1928). On the use and interpretation of certain test criteria for purposes of statistical inference: Part I. Biometrika.

[CR46] O’Brien PC, Fleming TR (1979). A multiple testing procedure for clinical trials. Biometrics.

[CR47] Perquin MN, Yang J, Teufel C, Sumner P, Hedge C, Bompas A (2020). Inability to improve performance with control shows limited access to inner states. Journal of Experimental Psychology: General.

[CR48] Perugini M, Gallucci M, Costantini G (2014). Safeguard power as a protection against imprecise power estimates. Perspectives on Psychological Science.

[CR49] Pocock SJ (1977). Group sequential methods in the design and analysis of clinical trials. Biometrika.

[CR50] Pramanik, S., Johnson, V.E., & Bhattacharya, A. (2020). A modified Sequential Probability Ratio Test. arXiv Preprint. Retrieved from. https://arxiv.org/pdf/1811.08478.pdf 10.1016/j.jmp.2021.102505 PMC9053723 35496657

[CR51] Purcell BA, Heitz RP, Cohen JY, Schall JD, Logan GD, Palmeri TJ (2010). Neurally constrained modeling of perceptual decision making. Psychological Review.

[CR52] Ratcliff R (1978). A theory of memory retrieval. Psychological Review.

[CR53] Reckase, M.D. (1983). A procedure for decision making using tailored testing. In *New Horizons in Testing* (pp. 237–255): Elsevier.

[CR54] Rouder JN (2014). Optional stopping: No problem for Bayesians. Psychonomic Bulletin & Review.

[CR55] Rouder JN, Speckman PL, Sun D, Morey RD, Iverson G (2009). Bayesian t tests for accepting and rejecting the null hypothesis. Psychonomic Bulletin & Review.

[CR56] Rushton S (1950). On a sequential t-test. Biometrika.

[CR57] Savage LJ (1954). The Foundations of Statistics.

[CR58] Schnuerch M, Erdfelder E (2020). Controlling decision errors with minimal costs: the sequential probability ratio t test. Psychological Methods.

[CR59] Schnuerch, M., Heck, D.W., & Erdfelder, E. (2021). Waldian t tests: Sequential Bayesian t tests with controlled error probabilities. PsyArXiv Preprint. 10.1037/met0000492 35420855

[CR60] Schönbrodt FD, Wagenmakers E-J (2018). Bayes factor design analysis: Planning for compelling evidence. Psychonomic Bulletin & Review.

[CR61] Schönbrodt FD, Wagenmakers E-J, Zehetleitner M, Perugini M (2017). Sequential hypothesis testing with Bayes factors: Efficiently testing mean differences. Psychological Methods.

[CR62] Schumann, E. (2019). NMOF: Numerical optimization in finance (version 2.0-1). https://cran.rproject.org/web/packages/NMOF/index.html

[CR63] Simonsohn U (2015). Small telescopes: Detectability and the evaluation of replication results. Psychological Science.

[CR64] Stefan, A.M., Evans, N.J., & Wagenmakers, E.-J. (2020). Practical challenges and methodological flexibility in prior elicitation. PsyArXiv Preprint. 10.1037/met0000354 32940511

[CR65] Stefan AM, Gronau QF, Schönbrodt FD, Wagenmakers E-J (2019). A tutorial on Bayes Factor Design Analysis using an informed prior. Behavior Research Methods.

[CR66] Stojić H, Schulz EP, Analytis P, Speekenbrink M (2020). It’s new, but is it good? How generalization and uncertainty guide the exploration of novel options. Journal of Experimental Psychology: General.

[CR67] Strack F, Martin LL, Stepper S (1988). Inhibiting and facilitating conditions of the human smile: A nonobtrusive test of the facial feedback hypothesis. Journal of Personality and Social Psychology.

[CR68] Thompson, N.A. (2009). Utilizing the generalized likelihood ratio as a termination criterion. In *Proceedings of the 2009 GMAC Conference on Computerized Adaptive Testing*.

[CR69] Thompson, N.A., & Ro, S. (2007). Computerized classification testing with composite hypotheses. In *Proceedings of the 2007 GMAC Conference on Computerized Adaptive Testing*.

[CR70] Townsend JT, Ashby FG (1983). Stochastic Modeling of Elementary Psychological Processes.

[CR71] Vanpaemel W (2010). Prior sensitivity in theory testing: An apologia for the Bayes factor. Journal of Mathematical Psychology.

[CR72] Verhagen J, Wagenmakers E-J (2014). Bayesian tests to quantify the result of a replication attempt. Journal of Experimental Psychology: General.

[CR73] Wagenmakers E-J, Lodewyckx T, Kuriyal H, Grasman R (2010). Bayesian hypothesis testing for psychologists: A tutorial on the Savage? Dickey method. Cognitive Psychology.

[CR74] Wald A (1945). Sequential tests of statistical hypotheses. The Annals of Mathematical Statistics.

[CR75] Wald A, Wolfowitz J (1948). Optimal character of the sequential probability ratio test. The Annals of Mathematical Statistics.

[CR76] Wetzels R, Matzke D, Lee MD, Rouder JN, Iverson GJ, Wagenmakers E (2011). Statistical evidence in experimental psychology: an empirical comparison using 855 t tests. Perspectives on Psychological Science.

